# *Salvia miltiorrhiza* extracts protect against retinal injury in a rat glaucoma model

**DOI:** 10.3892/etm.2014.1632

**Published:** 2014-03-24

**Authors:** QI ZHU, GUANFANG SU, LILI NIE, CHENGUANG WANG, YUXI HE, XIN LIU

**Affiliations:** Department of Ophthalmology, The Second Hospital of Jilin University, Changchun, Jilin 130041, P.R. China

**Keywords:** glaucoma, *Salvia miltiorrhiza*, Danshen, traditional Chinese medicine, retina, retinal ganglion cell, neuroprotection

## Abstract

Glaucoma is a serious progressive degenerative disorder of the eye that leads to the continuous loss of retinal ganglion cells. Traditional Chinese medicine provides an important source for new drug screening and identification. The present study used *Salvia miltiorrhiza* (Danshen) extracts to examine the potential neuroprotective effects for the eye in a rat model of experimental glaucoma. The results of the study indicated that *Salvia miltiorrhiza* extracts were unable to prevent intraocular pressure increase in the laser-induced glaucoma model, but the treatment did reduce cell loss during glaucoma progression. Therefore, the results provide the basis for the development of a novel therapeutic agent that exhibits neuroprotective effects against glaucoma. In the future, further studies are required to purify the extracts and determine the effective bioactive components of *Salvia miltiorrhiza.*

## Introduction

Glaucoma is a serious progressive disorder of the eye that is found primarily in aged populations and causes a high percentage of blindness (1–3). Elevated intraocular pressure (IOP) is considered to be one of the important factors in the initiation of glaucoma, leading to the shrinkage of the optic nerve and the loss of retinal ganglion cells (RGCs). Cell loss is irreversible; thus, self-recovery or restoration of vision is not possible following blindness. Stem cell transplantation currently offers one possibility to treat the disease. However, for the RGC layer, transplantation is unable to produce sufficiently high numbers of integrated cells or specialized differentiation of the transplanted cells (1,4). Therefore, neuroprotection during the progression of glaucoma represents the main approach of the current therapies. Investigating new neuroprotective agents for the eye is critical.

A number of traditional Chinese medicines that are known to be beneficial to the eye have been studied in animal models of glaucoma. For example, *Lycium barbarum* Lynn extracts were shown to exhibit neuroprotective effects on RGCs in various diseases, including glaucoma (5,6). Danshen (*Salvia miltiorrhiza*) has also been administered for eye protection, particularly in aged populations (7,8). Thus, it is highly possible that this extract exhibits neuroprotective effects in the retina in progressive glaucoma. Therefore, the aim of the present study was to examine the potential neuroprotection of *Salvia miltiorrhiza* extracts on the retina in a rat model of glaucoma.

## Materials and methods

### Ethics

The study was approved by the Ethics Committee of Animal Research of the Department of Ophthalmology (Second Hospital of Jilin University, Changchun, China). Animal care guidelines were strictly followed for all experimental procedures in the study.

### Animal model

In total, 30 Sprague Dawley rats (gender, male; age, 4–5 months) were obtained from the Animal Experiment Center (Second Hospital of Jilin University) and maintained in conditions with a normal light cycle. The animals were housed with three rats per cage.

A total of 10 animals served as controls and were anesthetized with 50 mg/kg ketamine and 10 mg/kg xylazine, but did not undergo surgery (control group). For the glaucoma model, following anesthesia, the rats were subjected to occlusion of aqueous outflow by laser irradiation under a slit lamp. This procedure was performed twice on the left eye, leaving the right eye as the control.

For the 20 animals that underwent surgery, the surrounding lateral lines of the eye were occluded with 60–70 laser pulses at a power of 0.8 W (time duration of each pulse, 0.2 sec; size, 60 μm in diameter; diode laser output, 532 nm). The procedures were performed by two experienced surgeons and the model had been previously validated in preliminary experiments.

### IOP

IOP was examined constantly with an Icare^®^ Tonolab tonometer (Icare Finland Oy, Espoo, Finland) in order to select the animals with successful aqueous outflow occlusion. All 20 rats exhibited a clear IOP elevation as early as 24 h after surgery, with a baseline value of ~20 mmHg and an increase of 5–10 mmHg. The IOPs remained elevated for several days.

### Salvia miltiorrhiza extract feeding

The *Salvia miltiorrhiza* extracts were obtained from Tianjin Tasly Pharmaceutical Co., Ltd. (Tianjin, China) and were administered to rats orally at 1 g/kg/day, starting 1 week prior to the glaucoma model experiment. The IC_50_ value of the extracts was 178 g/kg, based on preliminary experiments.

Of the 20 animals subjected to surgery, 10 animals were treated with *Salvia miltiorrhiza* extract (treatment group) and 10 animals were left untreated (glaucoma group).

### FluoroGold™ (FG; Fluorochrome, Denver, CO, USA) labeling for RGC quantification

Labeling of the rat retinas was performed one month following surgery. Retrograde labeling on the superior colliculus was achieved with the application of 5 μl 5% FG (Fluorochrome, Inc., Denver, CO, USA) soaked in gel foam. After six days, the animals were sacrificed and the retinas were obtained and mounted flat for cell counting (double-blindly). A total of eight random regions of the retina were selected for counting and six animals from each group were used for this experiment.

### Statistical analysis

Data are presented as the mean ± SD and were analyzed with SPSS software, version 17.0 (SPSS, Inc., Chicago, IL, USA). For statistical analyses, the t-test was performed and P<0.05 was considered to indicate a statistically significant difference.

## Results

### Salvia miltiorrhiza extract does not prevent IOP increase

**Figure 1:**
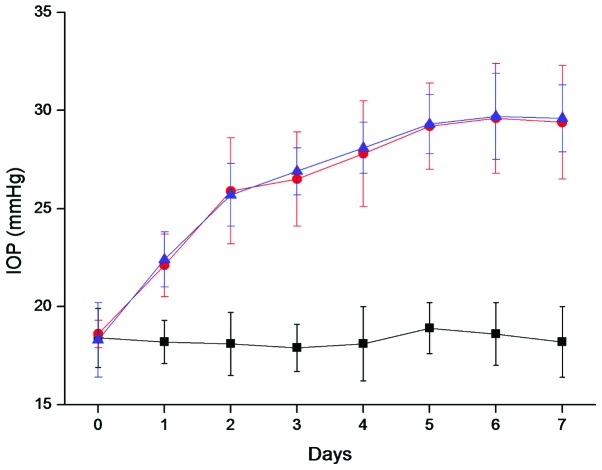
IOP changes in rats subjected to glaucoma-inducing surgery. There were no significant differences in IOP between the glaucoma and treatment groups, but these two groups showed a significant increase in IOP compared with that in the control group. Black, control; red, glaucoma group; blue, treatment group; IOP, intraocular pressure.

Results following surgery indicated that there was a rapid and steady increase in IOP for 7 days in the left eyes of the rats subjected to aqueous outflow occlusion, demonstrating the success of the glaucoma model. However, the oral administration of *Salvia miltiorrhiza* extract for one week did not prevent the IOP increase in the treatment group (Fig. 1). This indicates that *Salvia miltiorrhiza* extract does not change the physiological circulation properties of the aqueous outflow and is unable to decrease the IOP.

### Salvia miltiorrhiza extract protects RGCs

**Figure 2:**
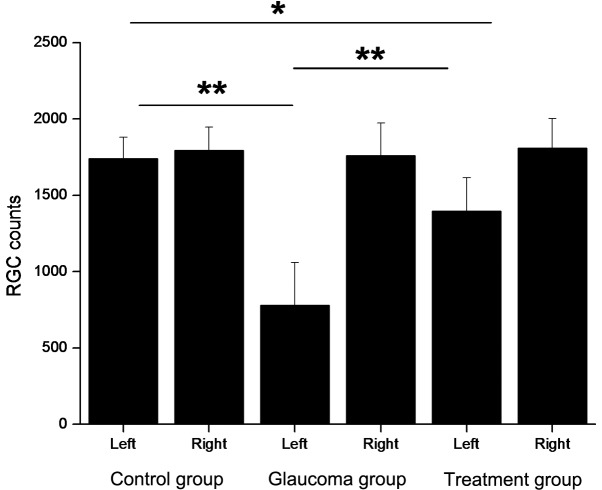
Oral administration of *Salvia miltiorrhiza* extract protects RGCs (counts per mm^2^). RGC loss in the left eye of the rats decreased significantly in the treatment group compared with that in the glaucoma group. ^*^P<0.05; ^**^P<0.01 for the difference between the left eyes indicated by the horizontal line ends. RGCs, retinal ganglion cells.

The results indicated that even with a rapid increase in IOP, orally administered *Salvia miltiorrhiza* extract protected against RGC loss 30 days following the IOP increase. In the glaucoma group, the loss of RGCs in the left eye was statistically significant (P<0.01), while in the treatment group there was mild reduction in the number of RGCs in the left eye (P<0.05), which was statistically significant from that in the glaucoma group (P<0.01; Table I and Fig. 2).

## Discussion

Glaucoma-induced retinal degeneration and vision loss is a severe problem in Asian countries, particularly in aged populations. Various efforts have been made to improve the survival of retinal neurons following an IOP increase, including infusion of trophic factors, electrical stimulation and visual training (9–11). Extracts from traditional medicine, particularly herbal medicine, have been shown to improve the survival of RGCs in various models of neuronal injury, including glaucoma-induced cell loss (12,13). The present study employed *Salvia miltiorrhiza* extract feeding in a rat model of glaucoma to investigate the potential protective effects of this extract in glaucoma. The results demonstrated that the orally administered extract did not prevent the IOP from increasing, but did protect the RGCs from pressure-induced cell death.

*Salvia miltiorrhiza* (Danshen) has been widely used in formulations and has demonstrated anti-ageing effects, as well as neuroprotection (7,8). In addition, *Salvia miltiorrhiza*, as part of a formulation, has been used to reverse diabetes-induced retinopathy or glaucoma-associated retinal degeneration (7,14,15). The present study used *Salvia miltiorrhiza* extracts alone and further demonstrated the protective functions of these extracts on RGCs. *Salvia miltiorrhiza* is composed of numerous bioactive components, including danshensu (DSS), protocatechuic aldehyde, salvianolic acid B, cryptotanshinone and tanshinone. DSS is considered to be one of the most important components and may be partially responsible for the neuroprotective effects observed in the retina (16). Future studies are required to investigate the bioactive components of *Salvia miltiorrhiza* and determine whether purified DSS at various doses exhibits neuroprotective effects in glaucoma and other eye diseases.

In summary, the present study has demonstrated the neuroprotective effects of *Salvia miltiorrhiza* extracts on RGCs in a rat model of glaucoma. Thus, *Salvia miltiorrhiza* may be a new therapeutic agent for the clinical management of glaucoma.

## Figures and Tables

**Table I:** Orally administration of *Salvia miltiorrhiza* extract protects RGCs (counts per mm^2^, mean ± SEM).

Eye	Control group	Glaucoma group	Treatment group
Left	1,740±140	780±280^a^	1,396±220^b^,^c^
Right	1,794±153	1,760±214	1,810±195

a P<0.01 and

b P<0.05 vs. control group;

c P<0.01, vs. glaucoma group.

RGCs, retinal ganglion cells; SEM, standard error of the mean.
